# Glycine Regulates Neural Stem Cell Proliferation During Development *via* Lnx1-Dependent Notch Signaling

**DOI:** 10.3389/fnmol.2019.00044

**Published:** 2019-02-18

**Authors:** Abdelhamid Bekri, Meijiang Liao, Pierre Drapeau

**Affiliations:** ^1^Research Center of the University of Montreal Hospital Center (CRCHUM), University of Montreal, Montreal, QC, Canada; ^2^Department of Biochemistry, University of Montreal, Montreal, QC, Canada; ^3^Department of Neuroscience, University of Montreal, Montreal, QC, Canada

**Keywords:** LNX1, NSCs, glycine signaling, neurogenesis, Notch activity

## Abstract

During development of the zebrafish embryo, glycine signaling promotes the differentiation of neural stem cells (NSCs). We found that glycine signaling suppresses the expression of Ligand of Numb X1 (*lnx1*, Ligand of numb protein-x1), a gene of unknown function during NSC differentiation that is selectively expressed in the embryonic central nervous system (CNS). As a consequence, Numb levels were stabilized and Notch activity (measured as *her4.1* expression) was reduced, promoting NSC differentiation. These consequent actions were blocked by knockdown of *lnx1*. In contrast, *lnx1* overexpression increased NSC proliferation and led to defects of neural tube closure at the early stages of development. Thus, our data provide evidence that glycine/*lnx1* signaling modulates NSC proliferation by regulation of Notch signaling.

## Introduction

During neuronal development an early spontaneous electrical activity is generated in neural stem cells (NSCs) as an essential step for their proliferation, migration and differentiation (Spitzer, 2006) and involves several neurotransmitters including glutamate, GABA and glycine (Demarque et al., 2002; Scain et al., 2010). Here we investigated the role of glycine signaling during neuronal development in the zebrafish embryo. We demonstrated previously that glycine signaling regulates NSC proliferation (Mcdearmid et al., 2006) and differentiation (Cote and Drapeau, 2012) by promoting survival of a subpopulation of NSCs (Bekri and Drapeau, 2018). An RNA sequencing analysis revealed that glycine signaling regulates several pathways in NSC development (Samarut et al., 2016) as well as some outlying genes, with Ligand of numb protein-x1 (*lnx1*) among the most affected.

*Lnx1* protein is a RING-type E3 ubiquitin ligase (De Bie and Ciechanover, 2011; Flynn et al., 2011) that degrades Numb (Dho et al., 1998), a cell fate determinant (Uemura et al., 1989). Furthermore, Numb is associated with Shh signaling (Di Marcotullio et al., 2011) and P53 signaling (Colaluca et al., 2008), both participating in glycine-dependent neurogenesis in zebrafish models (Samarut et al., 2016; Bekri and Drapeau, 2018). Importantly, Numb is well-known to be an inhibitor of Notch signaling (Roegiers and Jan, 2004; Mcgill et al., 2009), but further elucidations are required to understand how Notch and *lnx1* activity correlates with other pathways to fine-tune neuronal development.

We report here that glycine signaling suppressed *lnx1* expression in NSCs and consequently modulated Notch activity by controlling Numb protein degradation.

## Materials and Methods

More information about materials and methods is provided in Supplementary Materials.

### Zebrafish

Zebrafish (*Danio rerio*) were maintained at 28°C under a 12-h light/dark cycle in the crCHUM Zebrafish Facility and they were raised and manipulated as per guidelines of the Canadian Council for Animal Care and protocol approved (N15018PDz) by the crCHUM ethics committee. To knockdown gene expression, embryos were microinjected with morpholino (MO) as described previously (Bekri and Drapeau, 2018).

### FACS and RT-qPCR

*Tg(GFAP:GFP)* embryos were injected with glycine receptor-MO (Glr-MO) or Ctrl-MO. At 20 hpf, GFAP-NSCs were sorted by FACS. Then, total RNA was extracted and gene expression was quantified as described previously (Samarut et al., 2016). Sequence of each primer was designed by Snapgene software^®^.

### Whole-Mount *in situ* Hybridization and Immunostaining

Embryos were injected with Glr-MO or Ctrl-M, then subjected to *in situ* hybridization or immunostaining as described previously (Bekri and Drapeau, 2018).

### Western Blotting

Embryos were injected with *lnx1-6myc* or *gal4* mRNA, then total protein was extracted at desired stages. Western blotting was performed as previously described (Swaminathan et al., 2018).

### Probes and mRNA Synthesis

To make probes or mRNA, total RNA was extracted from 24 h post fertilization (hpf) of zebrafish embryos. Total RNA was reverse transcribed to cDNA. Then, used to make probes and full length *lnx1* as described previously (Brustein et al., 2013).

## Results

### Glycine Signaling Suppresses *lnx1* Expression and Regulates Neural Tube Development

**Figure 1:**
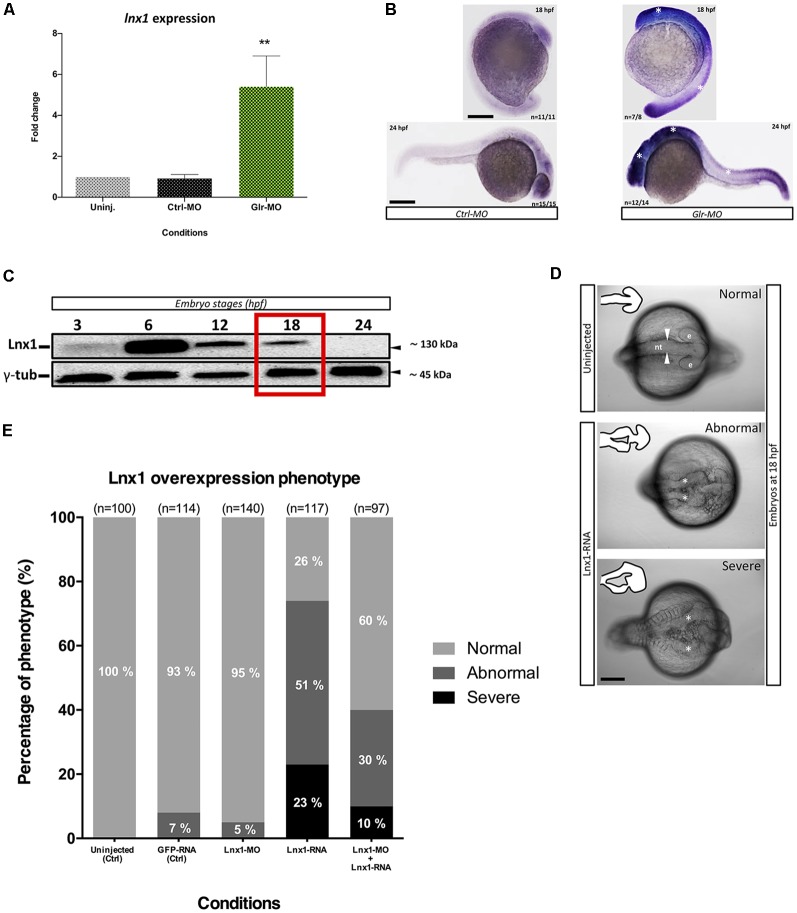
Glycine signaling regulates Ligand of numb protein-x1 (lnx1) expression during neural tube development. **(A)** Quantification of *lnx1* expressions into sorted GFAP^+^-neural stem cell (NSC) by RT-qPCR revealed a significant up-regulation of *lnx1* expression upon glycine signaling disruption compared with uninjected and Ctrl-morpholino (MO) conditions. One-way ANOVA statistical analysis was performed (*n* = 3, **p-value < 0.01). **(B)** Whole-mount *in situ* hybridization at 18 and 24 hours post fertilization (hpf) revealed that disruption of glycine signaling by glycine receptor morpholino (Glr-MO) induces an overexpression of *lnx1* into central nervous system (CNS; right) compared with control condition (left; Scale bar, 200 μm). **(C)** Time course of transient *lnx1* overexpression revealed by Western blot; embryos were injected with *lnx1-6myc* RNA then *in vivo* expression of *lnx1* protein was detected by anti-myc antibodies and followed during five-time point including, 3, 6, 12, 18 and 24 hpf, and anti-γ-tub antibody was used as loading protein control. **(D)** Neural tube closes defects upon *lnx1* overexpression; embryos were injected with *lnx1-6myc* RNA, then neural tube was imaged at 18 hpf. Phenotype of neural tube defect closure caused by *lnx1* overexpression was divided into three classes: normal neural tube (arrowheads), abnormal neural tube and neural tube with severe defects (asterisks) from top to down respectively. Structure of neural tube was delineated in the corner of each image. (e, eye; nt, neural tube. Scale bar, 250 μm). **(E)** Quantification of *lnx1* overexpression phenotype in each condition including uninjected, GFP-mRNA, *lnx1*-MO, *lnx1*-RNA or *lnx1*-MO and *lnx1*-RNA embryos.

We identified that expression of *lnx1* was strongly suppressed by glycine signaling during zebrafish development (Samarut et al., 2016). To confirm our transcriptomic study, we analyzed the expression level of *lnx1* upon disruption of glycine signaling by RT-qPCR and *in situ* hybridization. We used the *tg(GFAP:GFP)* line that expresses GFP under the *gfap* promoter (Bernardos and Raymond, 2006), which is an early marker of NSCs. Embryos from this line were treated with a Glr-MO to disturb glycine signaling, or with control Ctrl-MO or in uninjected eggs as control conditions. Embryos at 18 hpf were dissociated and GAFP^+^ NSCs were sorted, total RNA was extracted and *lnx1* expression was analyzed by RT-qPCR. Disruption of glycine signaling confirmed a significant increase of *lnx1* expression compared with Ctrl-MO or uninjected embryos condition (Figure 1A). To further validate these results, *lnx1* expression was visualized by whole-mount *in situ* hybridization, revealing a strong expression of *lnx1* upon Glr knockdown especially in the central nervous system (CNS) at 18 and 24 hpf stages (Figure 1B; right side, asterisk), compared with control condition which showed only a slight expression of *lnx1* in the brain (Figure 1B; left side). Taken together, these results confirm that glycine signaling suppresses *lnx1* expression into NSC at early stage of development.

We next tested the effects of early overexpression of *lnx1*. First, due to the unavailability of efficient antibodies against *lnx1*, we created a construct which expressed *lnx1* with myc-tag (*lnx1*-myc) to reveal *lnx1* expression by myc-tag antibodies. Then, we overexpressed *lnx1* by injecting *lnx1-myc* mRNA. Result showed a low expression level at 3 hpf and strong expression at 6 hpf, followed by degradation from 12 to 18 hpf until 24 hpf (midway through embryonic development), when* lnx1* expression was no longer detected (Figure 1C). Based on these results, we defined 18 hpf, near the start of neurogenesis, as the best time point to analyze the effect of early *lnx1* expression on zebrafish development. Control embryos showed normal brain and neural tube development (Figure 1D, in the top), whereas those injected with* lnx1* mRNA showed a major defect of neural tube closure, especially during head development (Figure 1D, in the middle and bottom, asterisk). We then tested several doses of *lnx1* mRNA and determined that 40 pg was the lowest dose that consistently produced an effect. We classified the defective neural tube phenotype into three classes: normal, abnormal and severe (Figure 1D). Control embryos uninjected or injected with *GFP* mRNA or *lnx1*-MO showed normal development of the neural tube (Figure 1E). However, upon *lnx1* mRNA injection, many of the embryos showed defective neural tube closure (Figure 1E). To verify whether the defect of neural tube closure was caused by overexpression of *lnx1* and was not an artifact caused by toxicity of mRNA injections, we tested for rescue of the defect of neural tube closure by co-injection of *lnx1* mRNA with *lnx1*-MO to block translation of *lnx1* mRNA. The results revealed a partial rescue, with a doubling of the normal phenotype and reduction by half in the two classes of defective phenotypes (Figure 1E). Taken together, these results provide evidence that overexpression of *lnx1* induced a defect of neural tube closure, accruing in a major malformation of the head region during zebrafish embryogenesis.

### Glycine/*lnx1* Signaling Regulates Notch Activity and NSCs Proliferation

**Figure 2:**
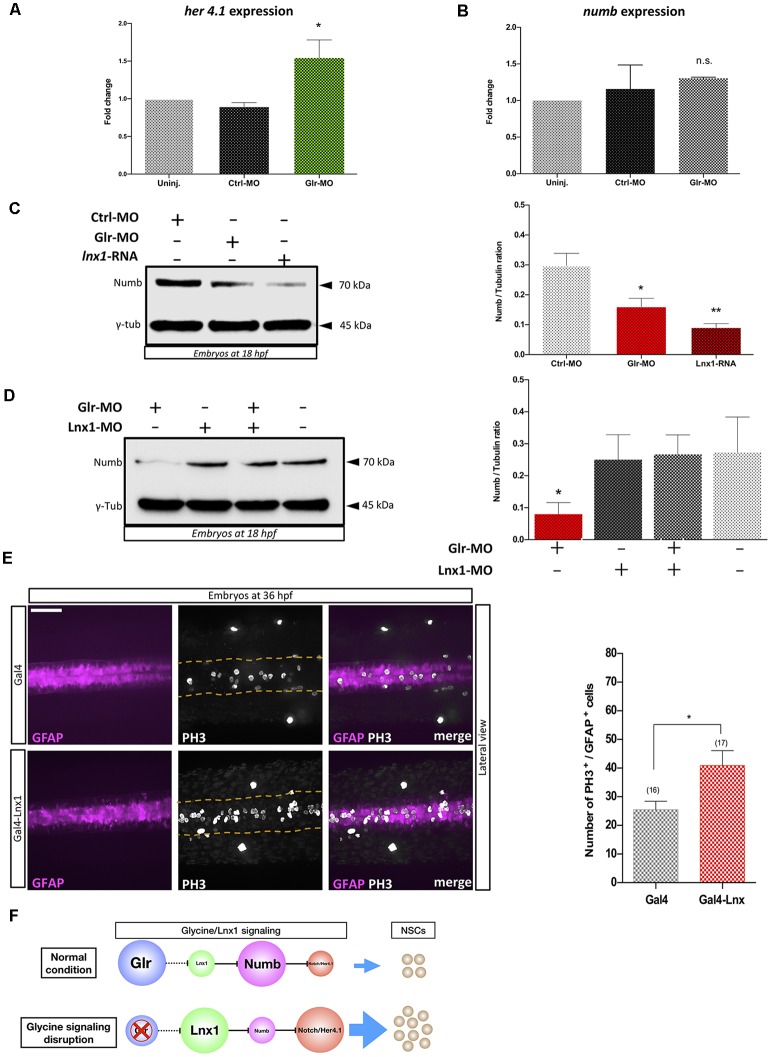
Glycine signaling modulates Notch activity *via* *lnx1* expression and promotes NSCs proliferation. Quantification of *her4.1* mRNA **(A)** and *numb* mRNA **(B)** level into sorted GFAP^+^-NSC by RT-qPCR revealed a significant increase of *her4.1* expression upon disruption of glycine signaling by Glr-MO compared with uninjected and Ctrl-MO conditions. However, no significant changing of numb expression. One-way ANOVA statistical analysis was performed [*n* = 3, *p-value < 0.05, not significant (n.s.)]. **(C)** Expression of Numb protein was revealed by western bolt at 18 hpf showing a significant degradation of numb protein upon disruption of glycine signaling by Glr-MO and overexpression of *lnx1* by *lnx1* mRNA injection compared with Ctrl-MO injections which were used as control condition. However, Co-injection of Glr-MO and *lnx1*-MO **(D)** rescued Numb protein degradation. One-way ANOVA statistical analysis was performed (*n* = 3, *p-value < 0.05, **p-value < 0.01). **(E)** Proliferation of GFAP^+^-NSCs (pink) in spinal cord by PH3 immunostaining (white) into tg(GFAP:Gal4, UAS:RED; top panel), and tg(GFAP:Gal4, UAS, *lnx1*, UAS:RED; bottom panel) revealed a significant increase of GFAP^+^-NSCs proliferation in *lnx1* overexpression embryos (right panel). One-way ANOVA statistical analysis was performed (*n* = 17, **p-value < 0.0001, scale bar, 250 μm). **(F)** The schematic model of regulation of Notch activity by glycine/*lnx1* signaling into zebrafish NSCs during early development.

*Lnx*1/2 are E3 ubiquitin ligases which promote the degradation of Numb and modulate Numb/Notch signaling during neurogenesis (Nie et al., 2002; Kageyama et al., 2007), though the role of *lnx1* in NSCs is unknown. To test whether disruption of glycine signaling in zebrafish NSCs, with elevated *lnx1* expression (Figure 1), modulates Notch signaling, we injected *Tg(gfap:GFP)* embryos at the one-cell stage with Glr-MO or Ctrl-MO, which were then sorted at 18 hpf GFAP^+^-NSCs, followed by RNA extraction. Using RT-qPCR we quantified *Her4.1* expression, a reporter of Notch activity in zebrafish (Takke et al., 1999). The results showed a significant increase of *her4.1* expression in GFAP^+^-NSCs upon glycine disruption, compared with uninjected and Ctrl-MO controls conditions (Figure 2A). This suggests that disruption of glycine signaling promotes Notch activity in NSCs.

We hypothesized that disruption of glycine signaling could modulate Numb protein expression, the main mediator between *lnx* and Notch signaling (Nie et al., 2002). We therefore used total RNA extracted from GFAP^+^-NSCs upon glycine signaling disruption to quantify *numb* expression by RT-qPCR. The results showed no significant change in *numb* mRNA level between disruption of glycine signaling (Glr-MO) and control conditions (Glr-MO or uninjected embryos) in NSCs (Figure 2B). However, analysis of Numb protein expression by western blot using anti-Numb antibody revealed a decrease in Numb protein level upon disruption of glycine signaling (Glr-MO) compared with control condition (Ctrl-MO; Figure 2C). This result suggests that while the *numb* mRNA level was unaffected by disruption of glycine signaling, Numb protein was degraded, likely *via* up-regulation of *lnx1* expression. To confirm that overexpression of *lnx1* in zebrafish embryos could mimic the disruption of glycine signaling and degrade Numb protein expression, we overexpressed *lnx1* and analyzed Numb protein expression at 18 hpf. The results showed an important decrease of Numb protein (Figure 2C). Finally, to verify whether degradation of Numb protein by glycine signaling was due specifically to *lnx1* overexpression, we tested whether down regulation of *lnx1* upon disruption of glycine signaling rescued Numb expression. To do so, we injected embryos with Glr-MO, *lnx1*-MO or both Glr-MO and *lnx1*-MO and evaluated Numb protein expression in each condition compared with uninjected embryos. The results showed a significant reduction of Numb protein level upon disruption of glycine signaling by Glr-MO compared with control whereas co-injection of Glr-MO and *lnx1*-MO together rescued the Numb protein level (Figure 2D). These results provide evidence that glycine/*lnx1* signaling modulates Notch activity by controlling Numb protein degradation in NSCs.

By analogy to *lnx2* (Won et al., 2015; Yin et al., 2015), we hypothesized that glycine/*lnx1* signaling controls NSC proliferation and that its disruption would cause a developmental phenotype with stabilized NSCs. To test this hypothesis, we expressed *lnx1* specifically in NSCs by generating a stable zebrafish line expressing *lnx1* (*UAS:lnx1*) in the *Tg(UAS:RFP)* reporter background, thus generating the double*-Tg(UAS:lnx1;UAS;RFP)* effector-line (Supplementary Figure S1A). First, to validate the transcriptional activation of *lnx1* in the *Tg(UAS:lnx1;UAS;RFP)* line, we induced ubiquitous expression of *lnx1* by injections of *Gal4*-activator mRNA (20 pg) into *Tg(UAS:lnx1;UAS:RFP)* or *Tg(UAS:RFP)* embryos, with the latter as controls. At 18 hpf, neural tube development was evaluated and *lnx1* mRNA level was analyzed by semi-quantitative RT-qPCR. The results showed a drastic defect of neural tube closure in *Tg(UAS:lnx1;UAS;RFP)* embryos compared with *Tg(UAS:RFP)* embryos, a phenotype similar to that of *lnx1* mRNA injection (data not shown). Moreover, quantification of *lnx1* mRNA levels demonstrated a strong transcriptional activity of *lnx1* in *Tg(UAS:lnx1;UAS;RFP)* compared with *Tg(UAS:RFP)* control. However, no significant change in transcriptional activity was observed in *rpl13a* and *ef1a*, used as reference genes (Supplementary Figure S1B,C). These results replicated the defect of neural tube closure observed by *lnx1* mRNA injections and confirmed the phenotype upon ubiquitous early expression of *lnx1* (Figure 1).

Next, in order to test the effect of *lnx1* overexpression on NSC proliferation, we specifically overexpressed *lnx1* in NSCs by crossing *Tg(UAS:lnx1;UAS:RFP)* with *Tg(GFAP:Gal4)* adult zebrafish. Embryos were fixed at 36 hpf and proliferation was assayed by PH3 immunostaining. The results revealed similar GFAP^+^-NSC populations (pink color) in both conditions including *Tg(GFAP:Gal4;UAS:lnx1, UAS:RFP)* and *Tg(GFAP:Gal4;UAS:RFP*; Figure 2E, in the left). However, *in vivo* overexpression of *lnx1* in NSCs in the *Tg(GFAP:Gal4;UAS:lnx1, UAS:RFP)* line revealed a large increase of PH3^+^-NSCs compared to the *Tg(UAS:RFP)* control line (Figure 2E, in the middle). This result indicates that early expression of *lnx1* in NSCs promotes their proliferation. Taken together, these results provide evidence that glycine/*lnx1* signaling modulates NSC proliferation through regulation of Notch activity (Figure 2F).

## Discussion

During neuronal development, several molecular changes take place in NSCs when glycine signaling is disrupted (Samarut et al., 2016). We demonstrated with different approaches that disruption of glycine signaling induced an overexpression of *lnx1* in NSCs (Figure 1). While regulation of *lnx2* transcription has been related to *Gli3* and *RunX2* (Pregizer et al., 2007; Wang et al., 2014), no transcription factors or pathways have been related to *lnx1* expression, leaving it as somewhat of an orphaned gene. However, increased *lnx1* expression reduces expression of the glycine transporter 2 (GlyT2) and impairs glycine transport in cortical neurons (Núñez et al., 2017). We showed that disruption of glycine signaling by knockdown of glycine receptors (Glrs) induced an overexpression of *lnx1* in NSCs. Furthermore, we demonstrated that GFAP^+^-NSCs up-regulated *lnx1* upon disruption of glycine signaling (Figure 1). Thus, glycine signaling suppresses *lnx1*, which appears to increase GlyT2, possibly as a homeostatic mechanism to regulate glycine levels. On other hand, a few studies have highlighted the potential role of the *lnx* protein family during developmental stages. Investigation of the Shh signaling component “Gli3” revealed that in knockout mice (Gli3^−/−^) there is an increased expression of *lnx2* and a dramatic decrease of Numb protein level in NSCs. These Gli3^−/−^ mice exhibit hydrocephaly and reduced cortical thickness as well (Wang et al., 2011, 2014). While *lnx2* signaling during neurogenesis is well explored, the role of *lnx1* in NSC development remained unknown. We demonstrated that transient expression of *lnx1* at an early stage of development caused a severe defect of neural tube closure in the head region, probably caused by the early loss of Numb proteins during embryogenesis. In support of our results, Numb^−/−^ null mice exhibit a severe defect in cranial neural tube closure and die around embryonic day 11.5 (E11.5; Zhong et al., 2000). These neural tube defects could be caused by disruption of neuronal development by affecting NSC proliferation.

We demonstrated that disruption of glycine signaling modulated Notch activity by increasing *her4.1* expression. While it did not affect *numb* transcription yet, it reduced the Numb protein level as a consequence of *lnx1* overexpression (Figure 2). We reported in zebrafish that disruption of glycine signaling increased NSC proliferation (Mcdearmid et al., 2006; Cote and Drapeau, 2012). Herein, by using a novel transgenic *Tg(UAS:lnx1;UAS;RFP)*, we showed that overexpression of *lnx1* in NSCs promotes their proliferation (Figure 2). These results provide compelling evidence that glycine signaling controls degradation of Numb *via* regulation of *lnx1* expression and modulate Notch activity and proliferation of NSCs. Over all, in this study we suggest that glycine/*lnx1* signaling controls NSC proliferation and differentiation by modulating the Notch pathway.

## Data Availability

All datasets generated for this study are included in the manuscript and the supplementary files.

## Author Contributions

AB conceived and performed most of experiments, generated the transgenic line with assistance from ML, wrote the manuscript. ML provided expertise and feedback. AB and PD reviewed and edited the manuscript. PD was responsible for supervision and funding acquisition.

## Conflict of Interest Statement

The authors declare that the research was conducted in the absence of any commercial or financial relationships that could be construed as a potential conflict of interest.
