# Supplementary material for: Glycine Regulates Neural Stem Cell Proliferation During Development via Lnx1-Dependent Notch Signaling
Source: Front Mol Neurosci. 2019 Feb 18;12:44. doi: 10.3389/fnmol.2019.00044 (PMC6387910; doi:10.3389/fnmol.2019.00044)
Supplement: Supplementary file 1 [file Data_Sheet_1.PDF]

Supplementary materials

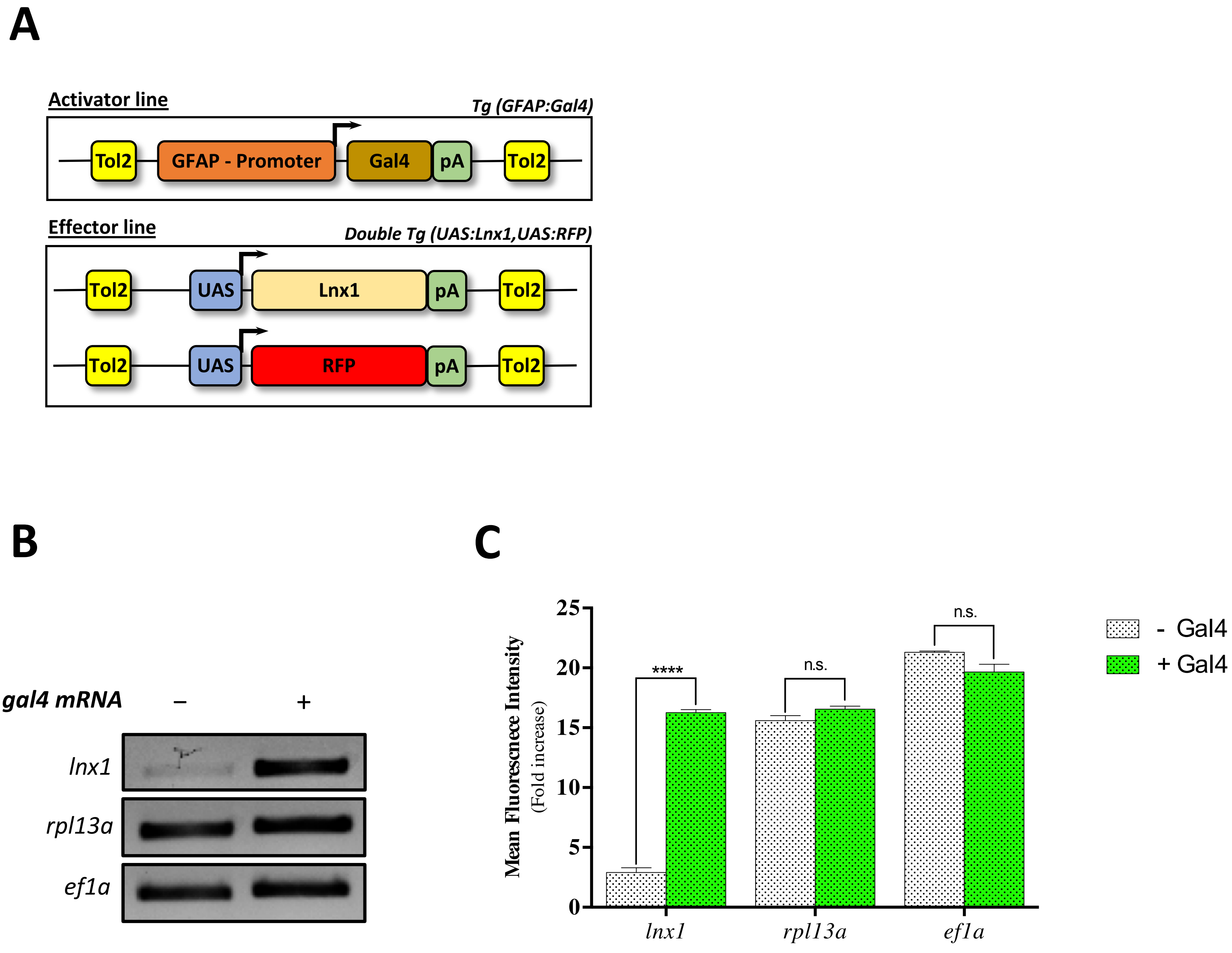

Figure S1. Generation and validation of a novel UAS:lnx1 transgenic line. (A) Schematic representation of the strategy to generate and activate *lnx1* transcription into $GFAP^{+}$-NSCs. (B) Transcriptional activation of *lnx1* into novel *tg(UAS:lnx1, UAS:RFP) by gal4* mRNA injection was visualised in the agarose gel, and showed a weak expression of *lnx1* without Gal4-activator comparted with injecting *gal4* mRNA condition. In contrast, reference genes including ef1a and rpl13a showed no changing of expression in both conditions. (C) Quantification of transcriptional activation of *lnx1* by semi-RT-qPCR reveled a significant overexpression of *lnx1* in *tg(UAS:lnx1, UAS:RFP)* upon activation by *Gal4* mRNA injection, compared with uninjected embryos. In contrast, no changing in reference gene expression including *ef1a* and *rpl13a* were noted. One-way ANVA statistical analysis was performed (n=3, **** p-value < 0.0001).

Supplementary materials

Transgenic line.

To generate the *UAS-lnx1* transgenic line *Tg(UAS:lnx1;UAS:RFP)*, first we cloned full length *lnx1* into *pME* donor plasmid by using *XhoI* and *XbaI* restriction sites. Then, we used Gateway Cloning® multisite technology to recombine four plasmids. The *pME-lnx1* middle donor vector was aligned with the 5' entry *p5E-UAS* and 3' entry *p3E-polyA*, then inserted into *pDestTol2CG2* destination vector. The *pTol2_UAS-lnx1* recombined construct was confirmed by sequencing. Second, *pTol2_UAS-lnx1* construct (20pg) was injected with transposase *Tol2* mRNA (30 pg) into the *Tg(UAS:RFP)* embryos at the one-cell stage. Finally, screening of the F0 generation was performed by outcrosses and selected embryos with green hearts which should contain transmission insertion was raised and the insertion of *lnx1* was confirmed by PCR.

FACS and RT-qPCR.

Tg(GFAP:GFP) embryos were injected at the one-cell stage with Glr-MO or Ctrl-MO. At 20 hpf embryos from each condition were dissociated and GFAP-NSCs were sorted by FACS and total RNA for each condition was extracted and *lnx1* expression was quantified by RT-qPCR. To generate statistical data, two independent experiments were performed, and PCR quantification experience was repeated three times for each condition. Two references genes (*polr2d* and *ef1a)* was selected for PCR quantification. Sequences of primers used for RT-qPCR were descried in (Supp. Table). To semi-quantitative PCR, *tg(UAS:lnx1,UAS:RFP)* embryos were injected by *gal4* mRNA. At 20hpf embryos were dissociated and total RNA was extracted. To made cDNA, 1 µg of RNA was reverse transcribed. Then, *lnx1* expression was evaluated by semiquantitative PCR as previously descried. Sequences of primers used for semi RT-qPCR were descried in (Supp. Table).

Whole-mount *in situ* hybridization and immunostaining.

Embryos were washed twice with phosphate-buffered saline 1X (PBS 1X) for 5 min each at room temperature and fixed overnight in cold 4% PFA. Embryos were then dehydrated in a gradient of methanol/PBS (25%, 50%, 75% and 100%) and stored in 100% ethanol at -20 °C. The embryos were then hydrated by inverted gradients and loaded into sample holders and mounted into an *in situ* hybridization device (Flogentec). Embryos were subjected for 22 hours to a continuous stream (1.7 ml/min) of successive reagents following the in situ hybridization protocol as previously described (Bekri et al., 2014). Finally, enzymatic detection was performed in PM purple reagent (Roche) under constant observation. To make immunostaining, *tg(GFAP-Gal4;UAS:lnx1;UAS:RFP)* embryos at 36hpf were washed twice by PBS 1X for 5 min each at room temperature and fixed overnight at +4°C in 4% PFA. Then, cell proliferation was assessed by immunostaining using primary antibody Anti-Phospho-Histone H3 and secondary antibody anti-rabbit conjugated with Alexa488.

Western blotting.

Embryos were injected with *lnx1-6myc* or *gal4* mRNA or GFP mRNA (as control), then total protein was extracted at desired stages. Western blotting was performed by using total protein extraction from two embryos in each condition, which was resolved on a 10% SDS-polyacrylamide gel. Then, a western blotting protocol was used as described previously (Swaminathan et al., 2018). To generate statistical data, three independent experiments were performed in each condition. Primary antibodies and dilution are as following: mouse, anti-myc, mouse, anti-NUMB and rabbit, anti- γ-Tub. Detection was performed using secondary goat anti-mouse and goat anti-rabbit conjugated with horse-radish peroxidase antibodies. Proteins were revealed by ECL Chemiluminescent reaction and imaged using ChemiDoc (Biorad). To quantify proteins, densitometry bands of three independent western blot images were analyzed by using Image Lab. Software (Biorad). The densitometry on protein bands for each protein was normalized to the appropriate loading control.

Supplementary materials

Probes and mRNA synthesis.

To make probes or mRNA, total RNA was extracted from 24 hours post fertilization (hpf) of zebrafish embryos. Then, total RNA was reverse transcribed to cDNA, which is used to amplify *lnx1* gene by PCR. To prepare *lnx1* probes a specific primer was designed to target around 1000 bp of *lnx1* coding sequence, then probes was prepared according to previous descriptions (Thisse and Thisse, 2008). Sequences of primers used to made probe were descried in (Supp. Table). Then, *lnx1* full-length was amplified by PCR and cloned in-frame of *6* x *myc* tag into the *pCS2-MT* plasmid. To make *lnx1-6myc* or *Gal4* mRNA, the *pCS2-lnx1-MT* or *pCS2-Gal4* were linearized by *NotI*. Then, 1µg of each linearized plasmid were used to synthesize *lnx1-6myc* or *Gal4* mRNA, respectively. *Sp6* transcription mMACHINE system (Ambion) was used to synthetize capped mRNA of each genes including, *lnx1-6myc* and *Gal4* mRNAs.

Supplementary materials

KEY RESOURCES TABLE

REAGENT	SOURCE	IDENTIFIER
Antibodies		
Anti-rabbit conjugated with Alexa488 (1:10 000)	ThermoFisher	Cat#R37116
Anti-Rabbit Conjugated to Horseradish Peroxidase (1:10 000)	Jackson ImmunoResearch	Cat#111-035-144
Gamma-Tubulin (1:1000)	Sigma	Cat#M4439
Myc-tag (1:1000)	ThermoFisher	Cat#13-2500
Numb (1:1000)	Santa cruz	Cat#sc-136554
Phospho histone H3 (1:500)	Millipore	Cat#06-570
Bacterial and Virus Strains		
DH alpha competent cells	Invitrogen	Cat#18265-017
Chemicals, Peptides, and Recombinant Proteins		
Anti-sens lnx1-RNA probes Conjugated to DIG	Synthesized	N/A
RNA extracted from zebrafish at 20hpf	This study	N/A
Protein extracted from zebrafish at 20hpf	This study	N/A
full length *lnx1-myc* RNA	Synthesized	N/A
full length *gal4* RNA	Synthesized	N/A
Critical Commercial Assays		
mMESSAGE mMACHINE SP6 kit	Ambion	Cat#AM1340
Superscript VILO cDNA synthesis kti	Invitrogen	Cat#11754050
LR Clonase™ II Plus enzyme	ThermoFisher	Cat#12538120
RevertAid First Strand cDNA Synthesis Kit	ThermoFisher	Cat#K1621
Experimental Models: Organisms/Strains		
Tubingen long wild type zebrafish (TL)	ZIRC	ZIRC_ID: ZL86
Tg(UAS:RFP) zebrafish line	ZFIN	ID: ZDB-TGCONSTRCT-151214-11
Tg(UAS:LNX1;UAS:RFP) zebrafish line	This study	N/A
Tg(GFAP:GAL4) zebrafish line	ZFIN	ID: ZDB-TGCONSTRCT-170119-1
Tg(gfap:GFP)	ZFIN	**ZFIN ID:** ZDB-TGCONSTRCT-070117-154
Oligonucleotides		
RT-qPCR primers for *lnx1, her4.1 and numb*<br>*ef1a*-F: 5'-CCTTCGTCCCAATTTCAGG-3'<br>*ef1a*-R: 5'-GGGTGGTTCAGGATGATGAC-3'<br>*rpl13a*-F: 5'-GCGGACCGATTCAATAAGG-3'<br>*rpl13a*-R: 5'-CTCCAGTGTGGCGGTGAT-3'.<br>*lnx1*-F2: 5'-TGGAGGATAACAGCCAATCC-3'<br>*lnx1*-R2: 5'-GTCCTGTCGAAGTTGCGATT-3'<br>*numb*-F: 5'-GGCACGGACACTCATTGTAG-3'<br>*numb*-R: 5'-TGACCGACCTTATTCATCTCC-3'<br>*her4.1*-F: 5'- CAGAGAAACTCTACTGACAAACAAGC-3'<br>*her4.1*-R: 5'- GCTGCTGTTGATTCGCTCT-3'	sigma	N/A
Primers for cloning lnx1: sequence in methods<br>*lnx1*-F3: 5'-GCATCATCTAATCCCTGATGG-3'<br>*lnx1*-R3: 5'-CCTCCAGCAGGTGGAAGAT-3'	sigma	N/A
Primers to make lnx1 probes<br>(*lnx1*-F1): 5'-GGTTGGGATTTACCCGTCTAT-3'<br>(*T7-Lnx1*-R): 5'-TAATACGACTCACTATAGGGTCCATGGTGCCTCCTGTAG-3	sigma	N/A
Lnx1-MO: 5'-TCAGGTTGCGTTTTGAGCCATCCAC-3	Gene Tools	http://www.gene-tools.com
Recombinant DNA		
pCS2	addgen	www.addgene.org
pCS2-MT	addgen	www.addgene.org
pCS2-lnx1-MT	This study	N/A
pCS2-lnx1	This study	N/A
Tol2Kit	Tol2kit	http://tol2kit.genetics.utah.edu/index.php/Main_Page
Software and Algorithms		
Volocity	Improvision-Perkin Elmer	http://www.perkinelmer.com
Imagelab	Biorad	http://www.bio-rad.com
Flogentec	Flogentec	http://www.flogentec.com/
LightCycler 480	Roche	https://lifescience.roche.com
